# Individual cardiovascular responsiveness to work-matched exercise within the moderate- and severe-intensity domains

**DOI:** 10.1007/s00421-021-04676-7

**Published:** 2021-04-03

**Authors:** Felipe Mattioni Maturana, Philipp Schellhorn, Gunnar Erz, Christof Burgstahler, Manuel Widmann, Barbara Munz, Rogerio N. Soares, Juan M. Murias, Ansgar Thiel, Andreas M. Nieß

**Affiliations:** 1 Sports Medicine Department, University Hospital of Tübingen, Tübingen, Germany; 2 Interfaculty Research Institute for Sport and Physical Activity, Eberhard Karls University of Tübingen, Tübingen, Germany; 3 Institute of Sports Science, Eberhard Karls University Tübingen, Tübingen, Germany; 4 Faculty of Kinesiology, University of Calgary, Calgary, Canada

**Keywords:** Cardiovascular, Cardiorespiratory, Exercise training, Individual response, Responders

## Abstract

**Purpose:**

We investigated the cardiovascular individual response to 6 weeks (3×/week) of work-matched within the severe-intensity domain (high-intensity interval training, HIIT) or moderate-intensity domain (moderate-intensity continuous training, MICT). In addition, we analyzed the cardiovascular factors at baseline underlying the response variability.

**Methods:**

42 healthy sedentary participants were randomly assigned to HIIT or MICT. We applied the region of practical equivalence-method for identifying the levels of responders to the maximal oxygen uptake (V̇O_2max_) response. For investigating the influence of cardiovascular markers, we trained a Bayesian machine learning model on cardiovascular markers.

**Results:**

Despite that HIIT and MICT induced significant increases in V̇O_2max_, HIIT had greater improvements than MICT (*p* < 0.001). Greater variability was observed in MICT, with approximately 50% classified as “non-responder” and “undecided”. 20 “responders”, one “undecided” and no “non-responders” were observed in HIIT. The variability in the ∆V̇O_2max_ was associated with initial cardiorespiratory fitness, arterial stiffness, and left-ventricular (LV) mass and LV end-diastolic diameter in HIIT; whereas, microvascular responsiveness and right-ventricular (RV) excursion velocity showed a significant association in MICT.

**Conclusion:**

Our findings highlight the critical influence of exercise-intensity domains and biological variability on the individual V̇O_2max_ response. The incidence of “non-responders” in MICT was one third of the group; whereas, no “non-responders” were observed in HIIT. The incidence of “responders” was 11 out of 21 participants in MICT, and 20 out of 21 participants in HIIT. The response in HIIT showed associations with baseline fitness, arterial stiffness, and LV-morphology; whereas, it was associated with RV systolic function in MICT.

## Introduction

Although the beneficial effects of exercise training on cardiovascular and metabolic health are well-established, it is widely accepted that a variety of factors such as exercise duration and intensity, age, etc., will determine the efficiency of the exercise training intervention (Gabriel and Zierath 2017; Mattioni Maturana et al. 2020).

Exercise training investigations often report considerable inter-individual variability with regard to changes in cardiorespiratory fitness [i.e., maximal oxygen uptake (V̇O_2max_)] in response to a given standardized dose of exercise (Ross et al. 2015; Williams et al. 2019). In relation to this, the HERITAGE study—a large scale investigation on the cardiometabolic adaptations to short-term exercise training—showed considerable variability in the cardiorespiratory response to exercise training, even in individuals of the same family and in monozygotic twins (Bouchard et al. 1999). However, even though heritability and genetics were found to account for a large percentage (i.e., ~ 50%) of the training response, there is still a large amount of variability that remains unexplained. In fact, recent research has questioned whether heritability is the main determinant of exercise training responsiveness (Marsh et al. 2020), suggesting that non- or low-responders to training may not exist provided that the training stimulus is appropriate (Lundby et al. 2017; Montero and Lundby 2017). In particular, recent studies have highlighted the role of exercise intensity (i.e., the metabolic stress based on the exercise intensity domain) (Iannetta et al. 2020a) and training protocols (i.e., continuous versus interval training) (Williams et al. 2019) as factors that might explain the inter-individual differences in “trainability”. Nevertheless, the extent to which the trainability of the aerobic system is primarily based on individual factors (e.g., population, initial fitness), or on the characteristics of the prescribed exercise regimen remains unclear.

The physiological mechanisms primarily associated with the increase in V̇O_2max_ after an exercise training intervention in previously untrained individuals are still debated. Whereas, some authors consider that central components of oxygen (O_2_) delivery are mainly responsible for the improvements in cardiorespiratory fitness (Montero and Lundby 2017), others have proposed that enhancements in the diffusion of O_2_ within the active tissues and intracellular changes within the muscles play a major role in this adaptation (Skattebo et al. 2020; Hargreaves and Spriet 2020). From an O_2_ perfusion (O_2_ delivery) perspective, adaptations to endurance training are mostly attributed to greater post-intervention cardiac output, which is mostly modulated by a larger stroke volume. This improved pumping capacity is associated with increases in left ventricular mass and function, which are linked to increments in blood volume and greater compliance of the heart (Wagner 2008; Moreira et al. 2020). In addition, peripheral vascular adaptations to exercise training result in improved vascular conductance (Green et al. 2017), thereby further contributing to the improvements in O_2_ delivery to the active tissues (Wagner 2008). Regarding the improvements in O_2_ diffusion (O_2_ extraction), putative mechanisms mainly include exercise training-related increases in mitochondrial mass, capillary density, and O_2_ diffusion capacity (Skattebo et al. 2020). Although different studies have examined the role of the above-mentioned mechanisms on the cardiovascular and metabolic adaptations to exercise training, no investigation has comprehensively examined the association between the improvements in cardiovascular markers and the increase in cardiorespiratory fitness. By evaluating the individual responses to exercise training of different intensity and mode (i.e., interval vs continuous, and severe vs moderate-intensity domain) would help in elucidating the mechanisms that control the adaptations to exercise training, as well as to advance the field of exercise training counselling, public health, and preventive medicine.

Herein, we analyzed the cardiorespiratory and cardiovascular effects of a six-week high-intensity interval training (HIIT, within the severe-intensity domain) or moderate-intensity continuous training (MICT, within moderate-intensity domain) exercise program in previously healthy sedentary participants. Our specific aims were to: (i) quantify the incidence of responders and non-responders to training within the moderate and severe exercise-intensity domains; and (ii) investigate cardiovascular and exercise training parameters at pre-training that might explain the inter-individual variability in the V̇O_2max_ response.

## Methods

### The iReAct study

**Fig. 1:**
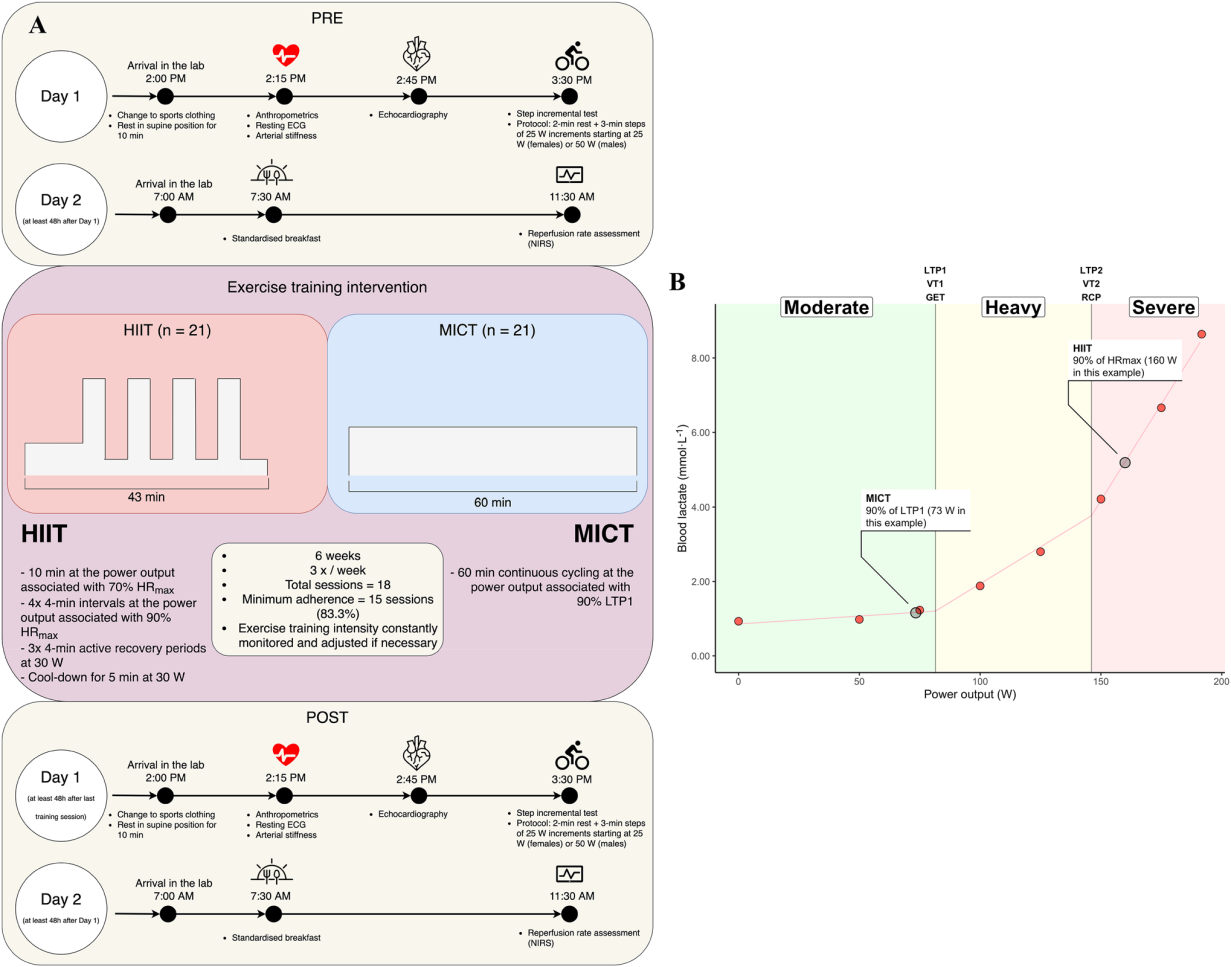
**a** displays a diagram of our experimental design. Note that baseline (PRE) and follow-up (POST) assessments were separated into three days of testing. Restrictions of minimum rest between days and last exercise training session are displayed under the day label within the white circles. Please, note that the time presented in each of the days are from a representative participant, as we had multiple participants per day. Therefore, although the times might not be the same for everyone, the time interval between each test were always equal.** b** displays an overview of acute exercise and exercise training prescriptions. A representative participant’s blood lactate response during the step incremental test is presented, displaying the exercise intensity thresholds analysis and the demarcation of exercise intensity domains. Please, note that the MICT (moderate-intensity continuous training) was performed within the moderate-intensity domain, the acute exercise within the heavy-intensity domain, and the HIIT (high-intensity interval training) within the severe-intensity domain. *LTP1* lactate turning point 1, *LTP2* lactate turning point 2, *VT1* ventilatory threshold 1, *VT2* ventilatory threshold 2, *GET* gas exchange threshold, *RCP* respiratory compensation point

The iReAct (Individual Response to Physical Activity) study was an interdisciplinary research project that investigated the physiological, affective, and cognitive responses to HIIT and MICT at the individual level (Thiel et al. 2020). Information pertaining the current study is presented below. Further details on the clinical trial can be found elsewhere (Thiel et al. 2020). In the present manuscript, we analyzed the effects of the first training period only. An overview of the research design and the outcome measures included in this manuscript is presented in Fig. 1a.

#### Recruitment and inclusion criteria

Participants (men and women between 20 and 40 years of age) were recruited primarily through the University of Tübingen and the University Hospital of Tübingen mailing list. Interested individuals were provided with detailed information regarding the study protocol and asked to fill out the European Health Interview Survey–Physical Activity Questionnaire (EHIS–PAQ) (Finger et al. 2015) to assess their physical activity levels. Participants that informed having less than 60 min per week of exercise during leisure time (including sports participation, aerobic activities, muscle strengthening) and no regular exercise training for the past six months prior to the study recruitment were eligible. Thereafter, potential participants were contacted by phone to verify their answers to the questionnaire. Subsequently, these potential participants were scheduled for a medical screening that included discussion of their medical history and a blood sample collection. Participants who presented a healthy status through the medical screening, who did not have a body mass index (BMI) greater than 30 kg m^−2^ throughout their life course, who had a current BMI between 18.5 and 30 kg m^−2^ or a percent of body fat within the normal range, and who did not present signs of anemia (due to iron deficiency) were eligible to start our diagnostics protocol. Further inclusion criteria related to the iReAct study included:

- Non-smokers;
- Currently not meeting the World Health Organization recommendations for moderate physical activity (less than 150 min/week);
- V̇O_2max_ between 25.0 and 50.0 mL kg^−1^ min^−1^ (measured on Day 1 of baseline measurements);
- No current or former eating disorder or obesity;
- No severe internal organs or neurological previous illness;
- No pregnancy or breastfeeding period;

### Included participants

A total of 58 participants were assessed for eligibility, 49 of whom were included in the randomization process and nine were excluded during medical diagnosis. Out of these nine excluded participants, seven were excluded for not meeting the inclusion criteria [iron deficiency anemia (*n* = 2), BMI above the predetermined upper limit (*n* = 1), under psychological treatment (*n* = 1), drug consumption (*n* = 1), and gastrointestinal issues (*n* = 2)], and two due to time management issues. During baseline measurements, five participants dropped-out due to time management issues (*n* = 1), withdrawal during the acute exercise test due to discomfort with the exercise (*n* = 1), lack of willingness to continue participation (*n* = 1), a migraine episode (*n* = 1), and lung condition being discovered (*n* = 1). Therefore, 44 participants engaged into the exercise training intervention (HIIT *n* = 22 and MICT *n* = 22). One participant in each group dropped-out during the exercise training intervention due to illness and not being able to complete the minimum adherence. A total of 42 participants (21 in each group) completed the study. Participants were informed of the experimental protocol and all associated risks prior to giving written informed consent. All procedures conformed to the Declaration of Helsinki and were approved by the Ethics Committee of the Medical Faculty at the University of Tübingen (882/2017BO1). An overview of participant’s characteristics can be found in Table 1.

**Table 1:** Participants characteristics on Day 1 of baseline measurements

	HIIT, *N* = 21^1^	MICT, *N* = 21^1^
Sex		
Females	14 (67%)	16 (76%)
Males	7 (33%)	5 (24%)
Anthropometrics		
Age (year)	26 ± 5	29 ± 6
BMI (kg/m^2^)	23.0 ± 2.9	24.2 ± 2.0
Height (cm)	171.3 ± 9.3	170.9 ± 8.6
Weight (kg)	67.8 ± 12.4	71.0 ± 8.9
Fitness		
Absolute V̇O_2max_ (L min^−1^)	2.16 ± 0.48	2.15 ± 0.36
Relative V̇O_2max_ (mL kg^−1^ min^−1^)	31.9 ± 4.1	30.4 ± 4.3
Blood markers		
Basophils (Tausd/µl)	0.04 ± 0.02	0.04 ± 0.02
Creatinine (mg/dl)	0.77 ± 0.17	0.71 ± 0.13
CRP (mg/dl)	0.22 ± 0.61	0.22 ± 0.36
Eosinophils (Tausd/µl)	0.16 ± 0.10	0.14 ± 0.13
Erythrocytes (Mio/µl)	4.77 ± 0.44	4.78 ± 0.41
Gamma-GT (U/l)	17 ± 13	20 ± 14
Glucose (mg/dl)	83 ± 9	81 ± 7
GOT (U/l)	13.76 ± 4.23	14.57 ± 4.46
GPT (U/l)	19 ± 9	22 ± 13
Hematocrit (%)	41.0 ± 3.5	40.2 ± 3.7
Hemoglobin (g/dl)	14.15 ± 1.28	13.83 ± 1.34
Leukocytes (1/µl)	6586 ± 1681	6249 ± 1357
Lymphocytes (%)	32 ± 8	31 ± 6
Lymphocytes (Tausd/µl)	2.04 ± 0.51	1.92 ± 0.40
Monocytes (%)	7.97 ± 1.56	7.68 ± 1.86
MPV (fl)	10.64 ± 0.73	10.52 ± 1.04
Neutrophils (Tausd/µl)	3.80 ± 1.51	3.67 ± 1.07
PDW (fl)	12.65 ± 1.54	12.54 ± 2.06
RDW (%)	12.37 ± 0.46	12.61 ± 0.72
Thrombocytes (Tausd/µl)	251 ± 55	248 ± 52

^1^ Statistics presented: *n* (%); mean ± standard deviation

*BMI* body mass index, *V̇O*_*2max*_ maximal oxygen uptake, *CRP* C-reactive protein, *Gamma-GT* gamma-glutamyl transferase, *GOT* glutamic-oxaloacetic transaminase, *GPT* glutamic-pyruvic transaminase, *MPV* mean platelet volume, *PDW* platelet distribution width, *RDW* red blood cell distribution width

### Experimental design

In the present study, all physiological variables were assessed before (PRE) and after (POST) the six weeks of exercise training. An overview of the experimental design is displayed in Fig. 1a. PRE and POST measures were always divided into two separate testing days.

#### Day 1

After arrival in the laboratory, participants were directed to the electrocardiogram (ECG) room, where they rested for 10 min in supine position. Thereafter, the resting ECG was conducted for 2 min with participants laying down in supine position (12-channel PC ECG, custo med GmbH, Ottobrunn, Germany), followed by the assessment of arterial stiffness (BOSO ABI system 100, BOSO, Jungingen, Germany), as previously described (Diehm et al. 2009). Participants were instructed to remain quiet for the full duration of this procedure. Then, anthropometrical measures were taken, which included height, weight, and a bioimpedance analysis (InBody770, InBody, Seoul, South Korea) for estimation of body composition (i.e., body fat and muscle mass). Height and weight measures were taken to the nearest 0.1 cm and 0.1 kg, respectively.

After these initial measurements, participants were accompanied to the echocardiography room, where they undertook a resting echocardiogram (Philips IE 33, Philips, The Netherlands). Afterwards, participants undertook a step-incremental test to volitional exhaustion on a cycle ergometer (Ergoselect 200, Ergoline GmbH, Bitz, Germany) for determination of V̇O_2max_, peak power output, and the lactate thresholds [lactate turning point 1 (LTP1) and lactate turning point 2 (LTP2)]. Before starting the test, baseline blood pressure and capillary blood lactate concentration ([La^−^]) were measured. The test began with a 2-min resting period on the bike, followed by 25-W step increments every 3 min, starting at 50 W for males and at 25 W for females, until task failure. [La^−^] was analyzed (Biosen S-Line, EKF, Cardiff, UK) by collecting capillary blood samples (20 µL) from the right earlobe during the last 20 s of each stage and immediately after volitional exhaustion. Blood pressure was measured again at 100 W, and immediately after volitional exhaustion. Heart rate and ECG were constantly monitored throughout the test (12-channel PC ECG, custo med GmbH, Ottobrunn, Germany). Breath-by-breath pulmonary gas exchange and ventilation were measured using a metabolic cart (MetaLyzer, CORTEX Biophysics, Leipzig, Germany). Calibration was performed before each test following the manufacturer’s instructions.

#### Day 2

48 h after *Day 1*, participants were instructed to come to the laboratory following at least nine hours of fasting (normally this ranged between 9 and 10 h). Upon arrival, a standardized breakfast was consumed (two cereal bars totalizing 160 kcal). Four hours after the breakfast, participants underwent a tissue oxygen saturation (StO_2_) reperfusion rate assessment in both lower and upper limbs using near-infrared spectroscopy (NIRS) (Portamon, Artinis Medical Systems, Netherlands). After a 10-min resting period on an examination table, skinfold measurements were taken in the areas where the NIRS probe was placed. Thereafter, a NIRS probe was placed on the belly of the *tibialis anterior* muscle for lower limb assessment. A pneumatic cuff (Flexiport; Welch Allyn Inc., Skaneateles Falls, NY, USA) was placed right below the knee (approximately 5 cm distal to the popliteal fossa) to induce limb blood flow when inflated. After a 2 min resting period (baseline period), the cuff was instantaneously inflated to 260 mmHg and maintained for 5 min (occlusion period). Once the 5 min of occlusion were reached, the pressure of the cuff was instantaneously released, and the NIRS signal was recorded for 8 more minutes (reperfusion period) (McLay et al. 2016a; b, c; Soares et al. 2018, 2019a). Once this measurement was completed, the NIRS probe was placed on the belly of the *flexor digitorum superficialis* muscle and the pneumatic cuff above the elbow (approximately 5 cm above the cubital fossa) for upper limb measurements. The same procedures as described above were repeated for baseline, occlusion, and reperfusion measurements. For all measurements (lower and upper limb), the NIRS probe was secured with a black elastic strap to avoid movement of the probe and covered with a black vinyl sheet to minimize the intrusion of external light. The raw data were collected at a frequency of 2 Hz (i.e., two samples per second). After exporting them, the data were averaged into 1-s bins. No data cleaning process was needed.

### Resting ECG and arterial stiffness

Resting ECG was recorded for two minutes, and the resting heart rate was calculated as the average response over this duration. Arterial stiffness was measured as the brachial-ankle index and the carotid-femoral pulse wave velocity, as previously described (Diehm et al. 2009; Baulmann et al. 2010).

### Echocardiography

Before and after the six-week training period, transthoracic echocardiography was performed with an iE33 ultrasound device from Philips (iE33, Philips Medical Systems B.V., Eindhoven, The Netherlands) with a 3.5 MHz transducer. The echocardiography was performed by three routine echocardiographic physicians. All measurements were done according to the standard recommendations and guidelines of the American Society of Echocardiography (ASE) and the European Society of Cardiology (ESC) (Lang et al. 2005; Nagueh et al. 2009, 2016; Rudski et al. 2010) to ensure high quality and valid data collection. All study participants were examined according to a standardized protocol.

To adjust the echocardiographic parameters to the cardiac cycle, a device-integrated ECG was recorded over the entire course of the study. The collected echocardiographic data were digitally stored and subsequently analyzed by the respective physician using the internal software. The storage of the collected data (including images and loops over five heartbeats) was achieved by image transfer to the department's internal software and additionally on DVD for a possible post-analysis.

The following measures were derived from the resting echocardiography.

#### Left ventricle and left atrium morphology

Left ventricular end-diastolic diameter (LVEDD), left ventricular mass (LV-Mass), LV-Mass index (LVMI), absolute heart volume, left atrium M-Mode (LA M-Mode), and left atrium size by planimetry (LA planimetric).

#### Right ventricle and right atrium morphology

Right ventricular end-diastolic diameter (RVEDD), and right atrium size by planimetry (RA planimetric).

#### Left ventricular systolic function

Fractional shortening (FS), ejection fraction by the Simpson method (EF Simpson), mean of mitral annular plane systolic excursion (MAPSE mean)—calculated as the mean between MAPSE septal and lateral, and mean of left ventricular excursion velocity (s’ LV mean)—calculated as the mean between s’ LV septal and lateral.

#### Left ventricular diastolic function

The ratio between the E-wave (i.e., mitral inflow velocity) and the A-wave (i.e., atrial inflow velocity) (E/A), ratio between the mean of diastolic mitral velocity (E’ mean)—calculated as the mean between the E’ septal and lateral—and the mean of diastolic atrial velocity (A’ mean)—calculated as the mean between the A’ septal and lateral (E’ mean/A’ mean), and the E/E’ ratio.

#### Right ventricular systolic function

Tricuspid annular plane systolic excursion (TAPSE), and right ventricular excursion velocity (s’ RV).

### Step incremental test to exhaustion

Breath-by-breath oxygen uptake (V̇O_2_) data were edited as follows: breath data points that were outside the 95% of confidence interval from the local mean were considered outliers and then removed. Thereafter, the data was interpolated on a second-by-second basis and averaged into 30-s bins for V̇O_2max_ analysis (Mattioni Maturana et al. 2018; Martin‐Rincon et al. 2019).

#### Maximal values

V̇O_2max_ was considered the highest 30-s V̇O_2_ average. V̇O_2max_ attainment was confirmed if at least two of the following three criteria were met, as per the American College of Sports Medicine guidelines (American College of Sports Medicine et al. 2018): (i) maximal heart rate within 10 beats per minute (bpm) of the maximal predicted value (220—age); (ii) a respiratory exchange ratio (RER) higher than 1.10; or (iii) a maximal [La^−^] of 8 mmol L^−1^. Peak power output (PO_peak_) was considered as the power output achieved at the moment of exhaustion and maximal heart rate (HR_max_) was considered as the maximal value achieved during the test. It is important to highlight that a verification ride for confirmation of V̇O_2max_ attainment was not performed, as this has been demonstrated that it does not add confidence to the V̇O_2max_ estimation and does not seem to be a robust and reliable measure (Murias et al. 2018; Wagner et al. 2021).

#### LTP1 and LTP2

Lactate thresholds were analyzed using a segmented regression model from which two breakpoints were estimated from the [La^−^]-power output relationship. LTP1 was determined as the first rise in [La^−^] above baseline levels (first breakpoint), which is accompanied by the first increase in V̇E in relation to V̇O_2_ (i.e., first ventilatory threshold) (Hofmann et al. 1997; Pokan et al. 1997; Binder et al. 2008; Hofmann and Tschakert 2017). LTP2 was determined as the second abrupt increase in [La^−^] (second breakpoint), which is accompanied by the second sharp increase in V̇E in relation to V̇O_2_ (i.e., second ventilatory threshold) (Hofmann et al. 1997; Pokan et al. 1997; Binder et al. 2008; Hofmann and Tschakert 2017). All these measures were analyzed as a function of power output, and then their corresponding V̇O_2_ values were analyzed from the V̇O_2_-power output relationship. Figure 1b shows an example of how lactate thresholds are calculated in a representative participant.

#### Efficiency slopes

The oxygen uptake efficiency slope (OUES), which is an index of cardiopulmonary functional reserve, was determined as the relationship between V̇O_2_ (in mL min^−1^) and V̇E (Baba et al. 1996; Hollenberg and Tager 2000; Onofre et al. 2017). OUES is analyzed as the slope of the following linear equation:

$$ {\dot{\text{V}}\text{O}}_{2} = a \times \log_{10} {\dot{\text{V}}\text{E}} + b $$

The ∆V̇O_2_/∆PO (V̇O_2_-power output relationship) slope, which is a surrogate of the efficiency of the aerobic metabolism to provide energy, was determined by linear regression using the least square method (Hansen et al. 1988; Prieur et al. 2005). The ∆HR/∆V̇O_2_ (heart rate- V̇O_2_ relationship) slope, which is an index of stroke volume and peripheral oxygen extraction, was also determined by linear regression using the least square method (Spiro et al. 1974; Fairbarn et al. 1994; Neder et al. 2001).

### StO_2_ reperfusion slope

The reperfusion rate was assessed as previously described (McLay et al. 2016c). Shortly, the baseline StO_2_ was calculated as the average of the last two minutes of the baseline period prior to ischemia. The StO_2_ reperfusion rate (slope 2) was calculated as the upslope of the StO_2_ signal during the first 10 s immediately after cuff release. Since the reperfusion rate presents a linear response within these 10 s, this allows for a simple slope calculation. The StO_2_ area under the curve was calculated as the total area under the reperfusion curve using the trapezoid method, above the baseline value until 4 min after cuff release was reached.

### Exercise training intervention

The HIIT and MICT prescription were designed with the goal that both interventions would be matched by energy expenditure (Andreato 2020). A literature search was performed to gather information on how exercise training interventions were usually prescribed in studies that reported their HIIT and MICT groups were matched (Rognmo et al. 2004; Helgerud et al. 2007; Tjonna et al. 2008; Molmen-Hansen et al. 2012; Mitranun et al. 2014; Ramos et al. 2016; Winn et al. 2018; Nie et al. 2018). After careful consideration, we prescribed the following exercise training programs (Fig. 1b displays an overview of the prescriptions):

### HIIT

The HIIT group performed 10 min of warm-up at the power output corresponding to 70% of their HR_max_, followed by four 4-min intervals at the power output corresponding to 90% of their HR_max_. Each high-intensity interval was interspersed with a 4-min active recovery at 30 W. After the last high-intensity interval a 5-min cool-down at 30 W was performed, totalizing 43 min of exercise. The power output at each percentage of HR_max_ was derived from the ∆HR/∆PO-relationship during the step incremental test performed on *Day 1*. To account for the delay in the heart rate response in relation to the increase in work rate in each step, the average of the last 30 s of each step was taken, and then plotted against power output, deriving the linear model used for the calculation. The exercise intensity at 90% of HR_max_ was also chosen as such intensity would be within the severe-intensity domain for this population (i.e., all the exercise intensities were above LTP2). Figure 1b displays an example in a representative participant.

### MICT

The MICT group performed 60 min of continuous cycling at the power output corresponding to 90% of LTP1. LTP1 was analyzed as described above, and such exercise intensity was prescribed for participants to cycle within the moderate-intensity domain (Binder et al. 2008; Hofmann and Tschakert 2017). Figure 1b displays an example in a representative participant.

#### Training monitoring

All exercise training sessions were performed on a cycle ergometer (ec5000, custo med GmbH, Ottobrunn, Germany) and participants’ heart rate and ECG were constantly monitored (3-channel ECG, custo med GmbH, Ottobrunn, Germany). After every training session, the exercise training data (i.e., second-by-second power output, cadence, and heart rate) were exported and stored for further processing. The heart rate data were cleaned using an anomaly detection algorithm to delete noisy data points (Dancho and Vaughan 2019). Each noisy data point was deleted, and the heart rate was then interpolated in a second-by-second basis. Once the training sessions of every week were completed (*n* = 3), the sessions were then ensembled-averaged and then averaged into 5-s bins. In this way, we retrieved one averaged dataset for each week. Thereafter, the weekly average response was compared with zones of smallest worthwhile difference around the prescribed heart rate. We defined these zones as ± 3 bpm from the prescribed heart rate to account for the day-to-day variability in exercise heart rate (Lamberts and Lambert 2009; Buchheit 2014). For the HIIT group, if the mean heart rate of the last two minutes of each high-intensity interval was below [90% HR_max_—3 bpm], the exercise intensity of the intervals was adjusted in + 5 watts for the following week. For the MICT group, if the mean heart rate across the sixty minutes of exercise was below [the heart rate associated with the prescribed exercise intensity—3 bpm], the exercise intensity was adjusted in + 5 watts for the following week. In case the heart rate response was above the smallest worthwhile difference, then the cycle ergometers were recalibrated by the manufacturing company. This training monitoring allowed to account for the changes in fitness throughout the weeks of training and, therefore, participants were always exercising within the originally prescribed relative intensity of exercise.

#### Minimum adherence

In order for participants to be included in the final analyses, a minimum of 15 out of the 18 prescribed exercise sessions had to be completed (minimum adherence = 83.3%). In case participants did not complete the minimum required, they were considered as dropouts.

### Training analyses

From the power output and heart rate data collected in each training session, the following was calculated:

#### Power output

We derived measures of power output in relation to peak power output (%PO_peak_), total work (power output × time), relative total work (total work normalized to body weight), total kcal (total work / 4.184), and relative total kcal (total kcal normalized to body weight). All the above measures were analyzed as a mean across the whole sessions, and there were also additionally calculated adjusted values for the HIIT (i.e., averages only considering the power output during the high-intensity intervals were calculated).

#### Heart rate

As aforementioned, prior to calculating the heart rate associated with each training session, the data were cleaned, interpolated on a second-by-second basis, and averaged into 5-s bins. Thereafter, the mean heart rate associated with every training session was derived (also expressed as a percentage of HR_max_ and heart rate reserve), as well as the iTRIMP (Manzi et al. 2009; Sanders et al. 2017).

### Responders’ classification

The responders’ classification was based on the ROPE + HDI decision rule (Kruschke 2018). This Bayesian decision-making method uses the region of practical equivalence (ROPE) in combination with the highest density interval (HDI) as the basis for accepting or rejecting the null hypothesis. The HDI summarizes the most credible values of a parameter (similar to the confidence interval in frequentist statistics), while the ROPE provides a range of values around the null value. Therefore, unlike null-hypothesis testing where values are tested against zero, the ROPE + HDI method calculates the percentage of HDI that is within the ROPE. Based on this percentage, there are different levels of significance, which we then apply to responders classification (see Fig. 4 for an overview and (Kruschke 2018) for an introduction to the topic).

#### Step 1—calculate the ∆V̇O_2max_ and its associated measurement error

The ∆V̇O_2max_ was analyzed as the raw difference (∆ = POST – PRE) from the absolute V̇O_2max_ response (L min^−1^). We additionally considered the technical error of measurement around the ∆V̇O_2max_ as the coefficient of variation associated with V̇O_2max_ measures (i.e., 5.6%), which also accounts for the random variation of true changes (Hecksteden et al. 2015, 2018). Therefore, the coefficient of variation was calculated as 5.6% of the baseline V̇O_2max_ for each individual, and a range around the ∆V̇O_2max_ was obtained:

$$ {\text{Measurement}}\;{\text{error}} = \frac{{{\text{Baseline}}\; \dot{V}O_{{2{\text{max}}}} \times 5.6\% }}{2}, $$

$$ {\text{Individual}}\;{\text{range}} = \left[ {\Delta \dot{V}O_{{2{\text{max}}}} - {\text{measurement}}\;{\text{error}};\Delta \dot{V}O_{{2{\text{max}}}} + {\text{measurement}}\;{\text{error}}} \right]. $$

Once the individual range was obtained, we then calculated the rough estimate of the standard deviation around the individual ∆V̇O_2max_:

$$ \Delta_{{{\text{sd}}}} = \frac{{{\text{Individual}}\;{\text{range}}}}{4}, $$

where ∆_sd_ is the individual rough estimate for the standard deviation around the ∆V̇O_2max_. The individual range is divided by 4 considering that approximately 99.9% of the data are within 4 standard deviations from the mean (∆V̇O_2max_ in our case) in a normal distribution.

#### Step 2—derive a normal distribution for each individual

Thereafter, once we have the mean (∆V̇O_2max_) and standard deviation (∆_sd_) from each individual, we could derive a normal distribution for each one of them (calculated from simulated 100 measures that spanned across all the possible ranges):

$$ f\left( x \right) = \frac{1}{{\sigma \sqrt {2\pi } }}e^{{ - \frac{1}{2}\left( {\frac{x - \mu }{\sigma }} \right)^{2} }} , $$

where $\sigma$ is the ∆V̇O_2max_, and µ is the ∆_sd_.

#### Step 3—calculate the HDI and ROPE

The HDI was retrieved from each individual normal distribution, calculated as 89% of the credible interval (Kruschke 2015, 2018). The ROPE was defined as the clinical relevant difference, calculated as the smallest worthwhile difference (Hopkins et al. 1999; Hecksteden et al. 2015, 2018; Williams et al. 2019; Ross et al. 2019). In practical terms, the ROPE was set as 20% of the baseline V̇O_2max_ standard deviation in both directions (i.e., ROPE = − 80 mL min^−1^ to 80 mL min^−1^. Full ROPE range = 160 mL min^−1^).

#### Step 4—responder classification

Each participant was then classified according to the HDI percentage within the ROPE. In the Bayesian framework, the percentage within the ROPE have different levels of significance (Makowski et al. 2019a). We then applied these levels labels to the responders’ classification. Participants that presented a negative ∆V̇O_2max_ were all considered as non-responders.

### Statistical analyses

Normality (Shapiro–Wilk’s test), homoscedasticy (Levene’s test), and multicolinearity were checked when applicable. A two-way analysis of variance was used to test main and interaction effects between- and within-subjects. In case of a significant main effect, to calculate differences between the exercise dose of HIIT and MICT a two-sample Welch’s *t*-test was performed. To calculate within-subject differences in the physiological outcomes (i.e., PRE vs POST) a paired Student’s *t* test was performed in each group (i.e., HIIT and MICT). To calculate between-subject differences of the outcome changes between the groups (i.e., ∆ HIIT ∆ vs MICT) a Games–Howell test (i.e., assuming non equal variances) was performed in each physiological outcome. The Bonferroni correction was applied for multiple comparisons. The alpha level was set at 0.05. To assess whether the regression to the mean phenomenon was present in the V̇O_2max_ results, we performed an analysis of covariance (ANCOVA), as previously suggested (Barnett 2004). Results presented are mean ± standard deviation unless otherwise stated.

#### Bayesian machine learning

For the regression analyses, we trained a Bayesian linear model (estimated using Markov chain Monte Carlo sampling with four chains of 2000 iterations and a warm-up of 1000) to find predictors that could explain the variability seen in ∆V̇O_2max_ in each one of the groups (HIIT and MICT) (Kruschke 2018; Makowski et al. 2019a). Potential predictors were set as variables at PRE from different domains: exercise training, performance, cardiac, and vascular measures. The normality of priors over parameters were checked, and the ROPE percentage was defined as the proportion of the posterior distribution within the [− 0.01, 0.01] range. The 89% credible intervals were based on HDI. Parameters were scaled by the mean and the standard deviation of the response variable. Effect sizes were labelled following Funder's recommendations (Funder and Ozer 2019).

All data analyses, editing, and visualizations were performed in R version 4.0.2 (R Core Team 2020) with the packages *tidyverse* (Wickham et al. 2019), rstanarm (Goodrich et al. 2020), *bayestestR* (Makowski et al. 2019b), *correlation* (Makowski et al. 2020), *igraph *(Csardi and Nepusz 2006), *ggraph *(Pedersen 2020), and *statsExpressions* (Patil 2019).

## Results

### Exercise training intervention

**Fig. 2:**
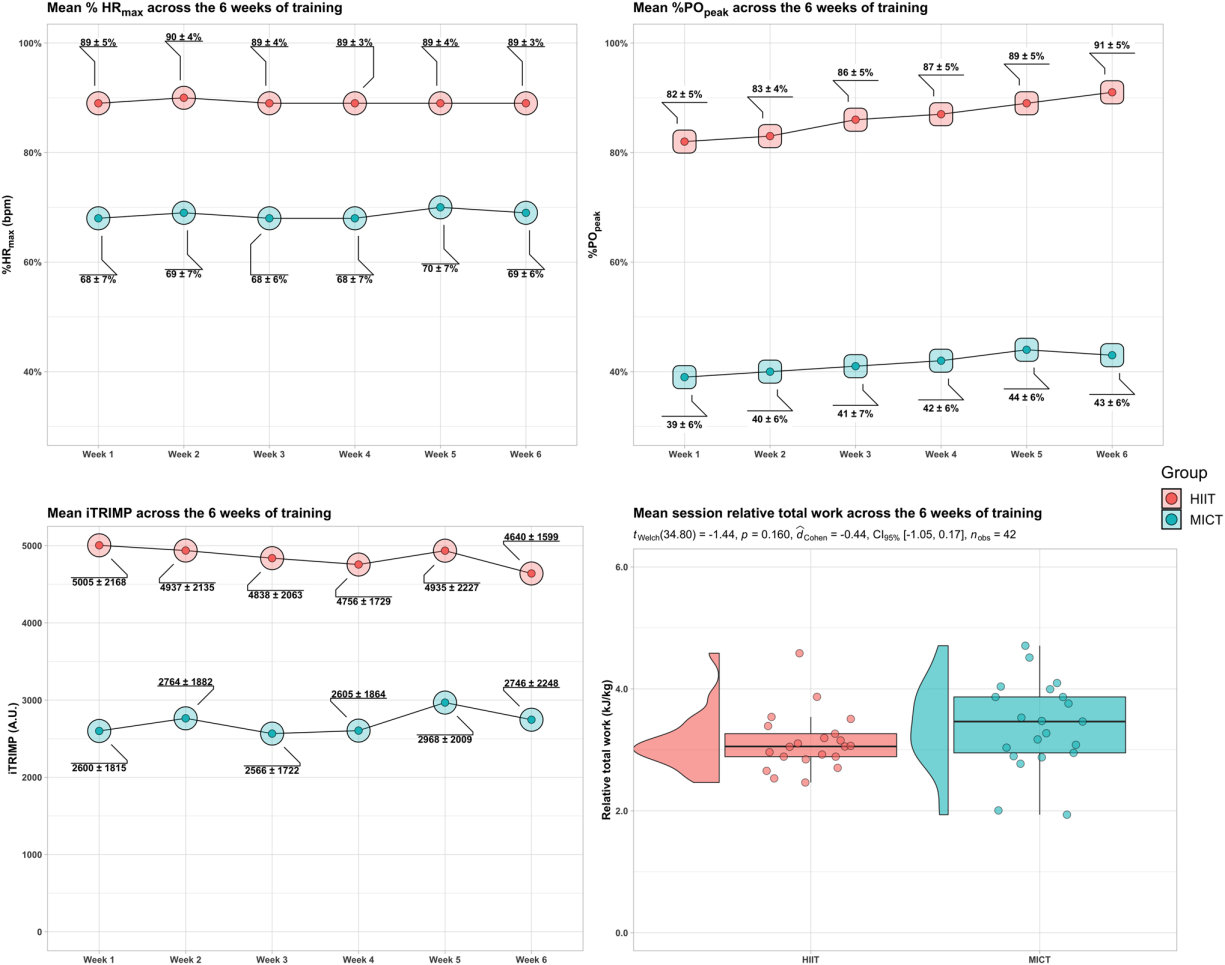
Left panels display heart rate measures over the six weeks of training. The upper left panel shows the %HR_max_ (percentage of maximal heart rate), and the lower left panel shows the iTRIMP (individualized training impulse). Right panels display measures of exercise dose. The upper right panel shows the %PO_peak_ (percentage of peak power output), and the lower right panel the mean relative total work for each group. Values are mean ± standard deviation. Please, note that %HR_max_ and %PO_peak_ values for the HIIT group are the data from the last minute of each high-intensity interval (see methods)

The relative total work (mean across sessions) associated with HIIT (3.13 ± 0.48 kJ/kg) and MICT (3.40 ± 0.72 kJ/kg) was not statistically different (*p* = 0.16; Cohen’s *d* = − 0.44, 95% confidence interval [− 1.05; 0.17]). The HIIT group increased the exercise intensity during the high-intensity intervals by 16 ± 6 W, and the MICT group by 8 ± 5 W. Mean values throughout the six weeks of exercise training are presented in Table 2. Figure 2 displays an overview of the mean values for % HR_max_, % PO_peak_, and iTRIMP of each week, as well as the mean relative total work across the 6 weeks of training.

**Table 2:** Mean values throughout the 6 weeks of exercise training in HIIT and MICT

	HIIT	MICT
%PO_peak_	50 ± 4 (86 ± 6)	42 ± 6
Absolute PO (W)	82 ± 15 (140 ± 27)	67 ± 15
Relative PO (W/kg)	1.22 ± 0.18 (2.09 ± 0.32)	0.94 ± 0.2
%HR_max_	75 ± 4 (89 ± 4)	69 ± 7
%HR_reserve_	60 ± 8 (82 ± 6)	48 ± 11
iTRIMP (A.U.)	4752 ± 1762	2710 ± 1512

HIIT values are presented considering the whole HIIT protocol, and considering only the high-intensity intervals between brackets

### Physiological adaptations

**Fig. 3:**
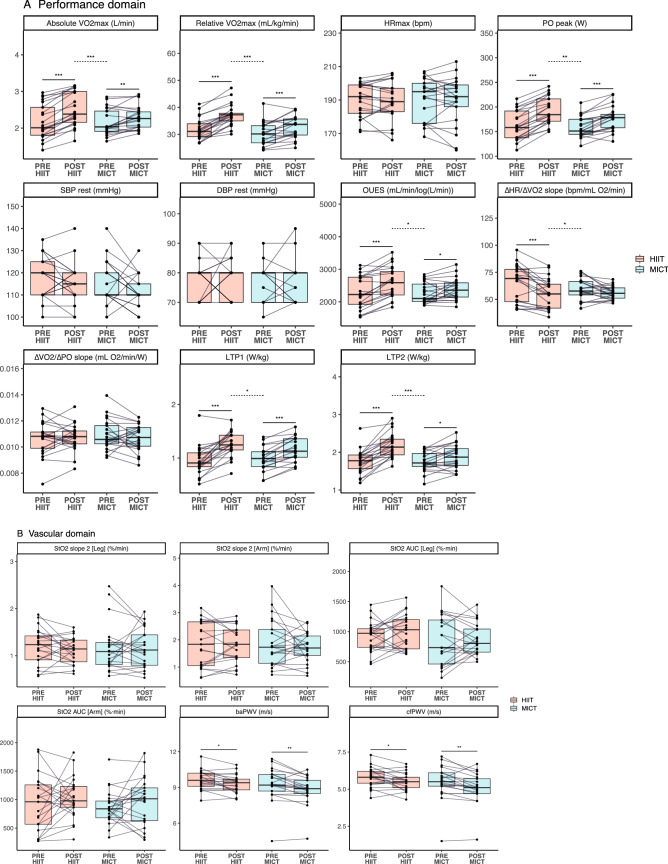

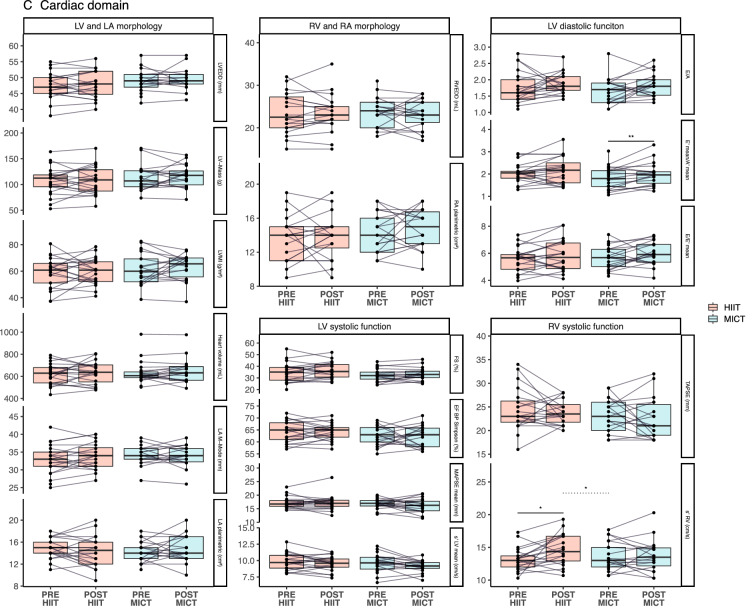
**a**–**c** Show the parameters of the performance, vascular, and cardiac domains, respectively. Values are shown for PRE and POST in each group. Continuous lines above the boxplots refer to within-group differences, and dotted lines refer to between-group differences. Note: * = *p* < 0.05, ** = *p* < 0.01, *** = *p* < *0.001*

Figure 3 displays the changes in the physiological outcomes of both groups (within- and between-group differences). HIIT resulted in a significant increase from PRE to POST in all the performance-related measurements (with the exception of HR_max_ and the ∆VO_2_/∆PO slope). In addition, an increase in systolic excursion velocity (i.e., s’ RV, right ventricular function, *p* < 0.05), and a decrease in arterial stiffness (i.e., baPWV and cfPWV, *p* < 0.05) were observed following HIIT. In the MICT group, there was a significant increase from PRE to POST in all the performance-related measures, with the exception of HR_max_ and the ∆HR/∆VO_2_ and ∆VO_2_/∆PO slopes. In addition, MICT presented a significant reduction in arterial stiffness (i.e., baPWV and cfPWV, *p* < 0.01), and an increase in E’ mean/A’ mean (*p* < 0.01), which indicates a greater LV-filling pressure. To investigate whether measures of LV-Mass were associated with high blood pressure, we correlated values of LV-Mass and systolic blood pressure. No significant correlations were found in MICT in both PRE (*r* = − 0.04, *p* = 0.5) and POST (*r* = 0.17, *p* = 0.9). In HIIT, the correlation at PRE was not significant (*r* = 0.37, *p* = 0.1), but a significant correlation at POST was observed (*r* = 0.46, *p* = 0.04). Between-group differences revealed a greater effectiveness of HIIT compared to MICT in improving absolute and relative V̇O_2max_ (p < 0.001), PO_peak_ (*p* < 0.01), lactate thresholds [LTP1 (*p* < 0.05) and LTP2 (*p* < 0.001)], OUES (ventilatory efficiency slope, *p* < 0.05), ∆HR/∆VO_2_ slope (*p* < 0.05), and systolic excursion velocity (i.e., s’ RV, *p* < 0.05).

### Responders’ classification

**Fig. 4:**
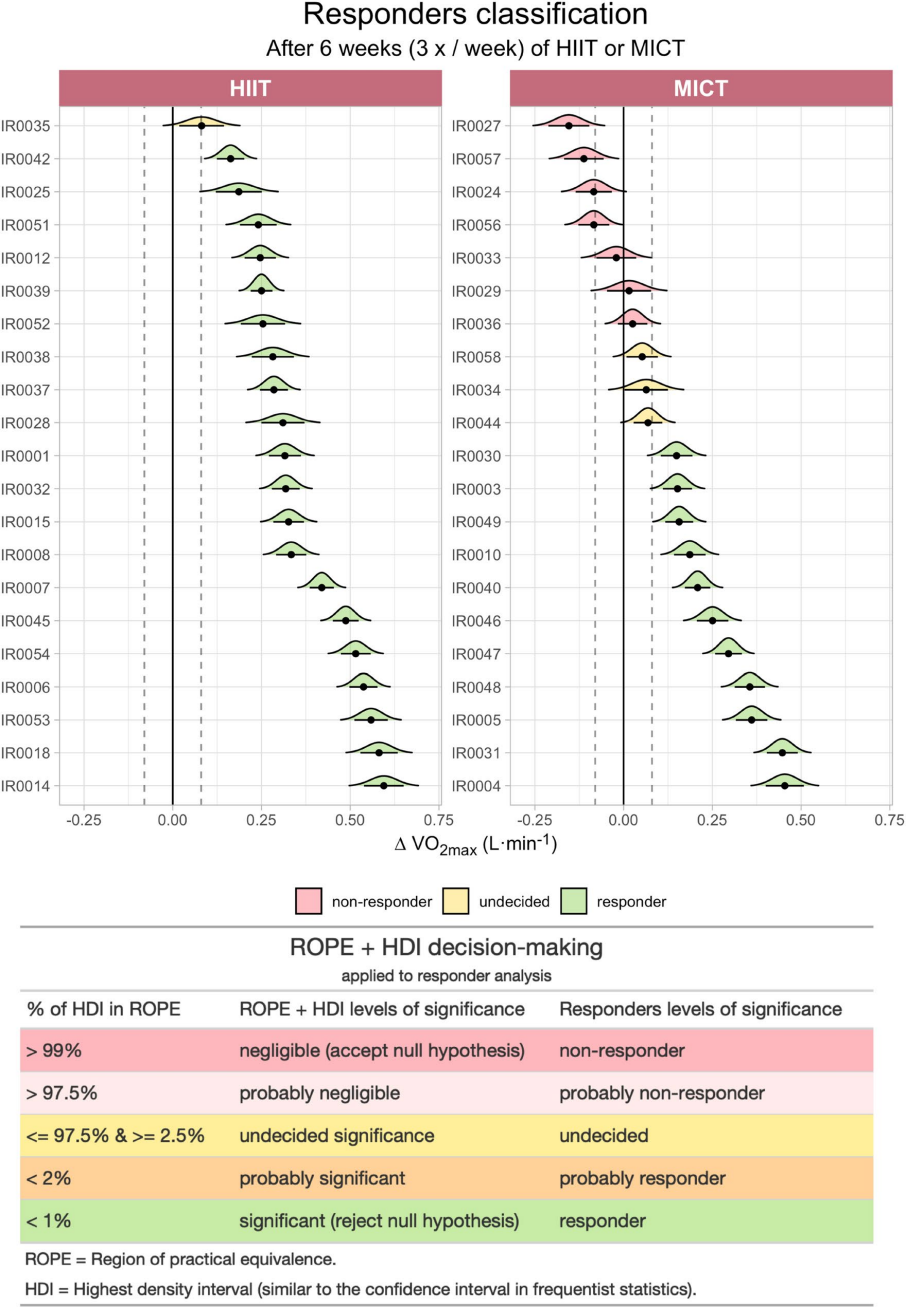
Overview of responders’ classification. The graph displays the ∆V̇O_2max_ (delta of maximal oxygen uptake) of each participant in HIIT (high-intensity interval training) and MICT (moderate-intensity continuous training). Black dots show the ∆V̇O_2max_, curves are the normal distribution curves derived for each individual given the measurement error around the ∆V̇O_2max_ value, horizontal black lines are the 89% HDI (highest density interval) derived from each curve, and vertical dashed lines around zero are the calculated ROPE (region of practical equivalence) (− 80 mL min^−1^ to 80 mL min^−1^). The table displays the levels of significance from the ROPE + HDI decision-making method applied to responders’ analysis. Each level is color-coded, and the three levels seen in the present sample (i.e., non-responder, undecided, responder) are displayed in the graph

All the participants achieved the minimum criteria for V̇O_2max_ attainment in both PRE (HR_max_ = 190 ± 12 bpm [7 ± 6 bpm within the predicted HR_max_], maximal RER = 1.26 ± 0.08, and maximal [La^−^] = 9.4 ± 1.8 mmol L^−1^) and POST (HR_max_ = 190 ± 11 bpm [8 ± 6 bpm within the predicted HR_max_], maximal RER = 1.27 ± 0.05, and maximal [La^−^] = 10.4 ± 1.9 mmol L^−1^). The summary of the individual responders’ classification as well as a summary of the decision-making process based on the statistical levels of significance can be found in Fig. 4. Even though a significant increase in V̇O_2max_ was observed in both HIIT (+ 17 ± 8%, *p* < 0.001) and MICT (+ 7 ± 9%, *p* = 0.003), the HIIT group showed a greater increase in V̇O_2max_ than MICT (*p* < 0.001). These results were confirmed by an ANCOVA, also showing a significant increase in V̇O_2max_ adjusted by baseline values (*ß* = 0.88, 95% CI = [0.76; 1.00], *p* < 0.001), and between-groups (*ß* = 0.22, 95% CI = [0.12; 0.32], *p* < 0.001). The HITT group presented only one “undecided” (5%) and 20 “responders” (95%), while the MICT group had seven “non-responders” (34%), three “undecideds” (14%), and 11 “responders” (52%). The changes in V̇O_2max_ for the HIIT group ranged from 81 mL min^−1^ to 595 mL min^−1^; whereas, the MICT group ranged from − 154 mL min^−1^ to 454 mL min^−1^.

### Cardiovascular markers at PRE associated with ∆V̇O_2max_

**Fig. 5:**
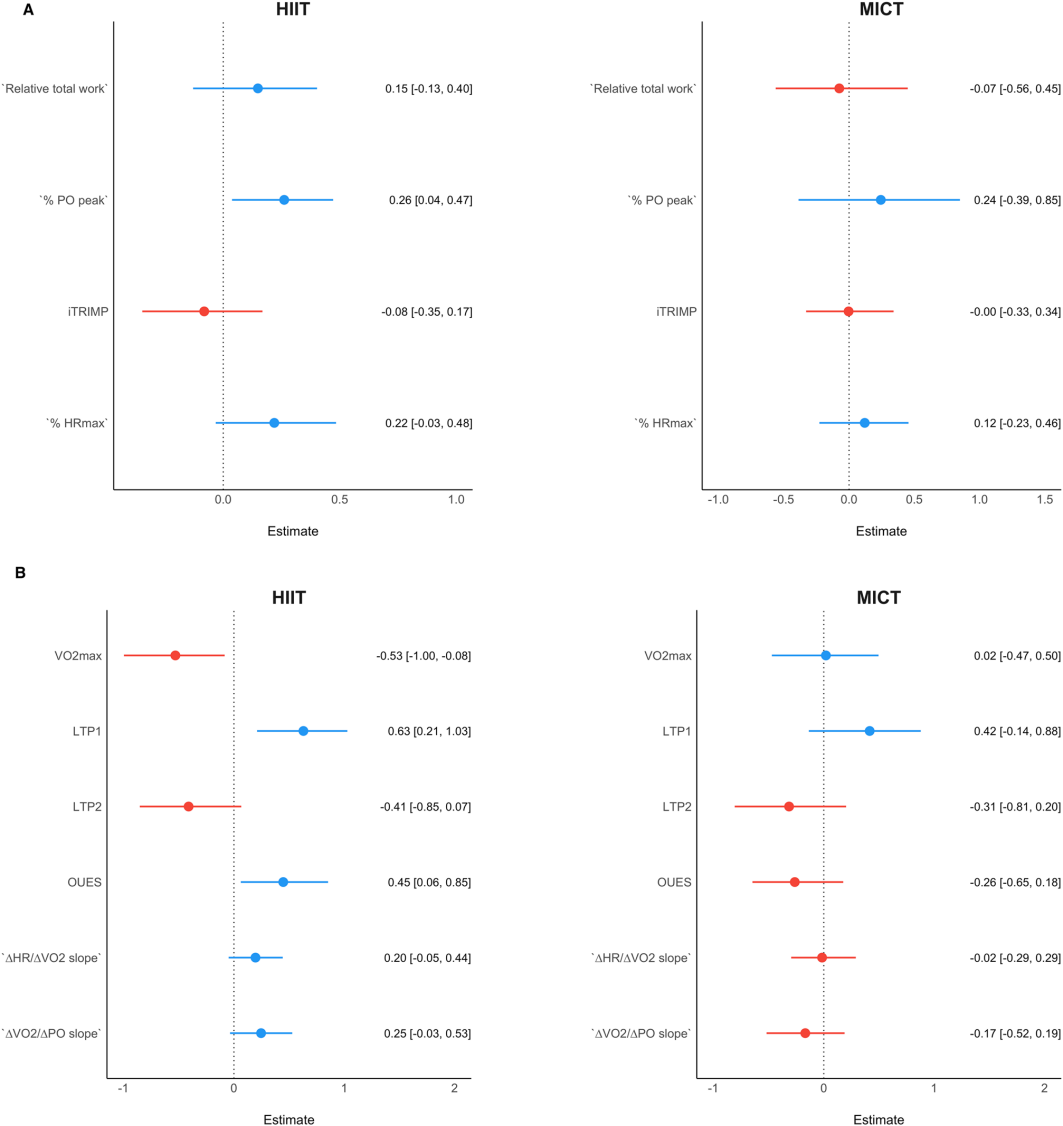

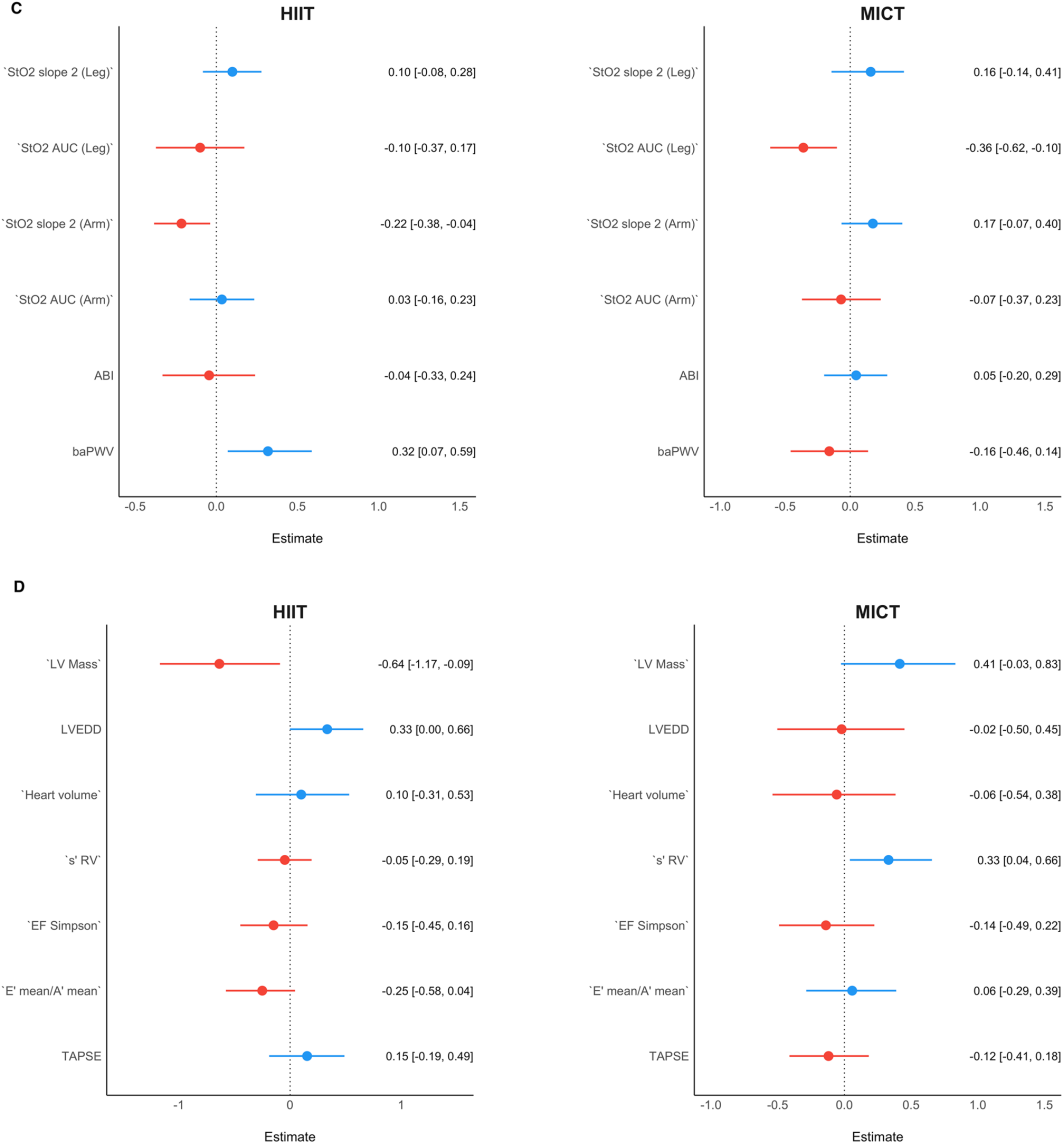
Each panel represents the summary of the Bayesian machine learning coefficients and their corresponding confidence intervals in each one of the domains in each group. These are values at baseline that could explain the variability in the ∆V̇O_2max_ in both HIIT and MICT. For example, if a variable shows a negative effect, it means that the greater this variable was at baseline, the lesser was the ∆V̇O_2max_ response after training, and vice-versa.** a** shows the summary of the exercise training domain results,** b** shows the summary of the performance domain results,** c** shows the summary of the vascular domain results, and** d** shows the summary of the cardiac domain results

Our Bayesian machine learning modelling indicated different potential variables that might have had an influence in the ∆V̇O_2max_ in both groups. Figure 5 displays a summary of the effect estimates and confidence intervals of each regression in each domain. Please, note that negative point estimates indicate that participants that presented a lower value for this given parameter, presented a higher ∆V̇O_2max_, and vice-versa.

In the exercise training domain, the exercise intensity as measured by the percentage of the peak power output (estimate = 0.26 [0.04, 0.47]) showed a significant influence in HIIT, such that participants that exercised in a higher percentage of peak power output showed a greater increase in V̇O_2max_.

In the performance domain, the trainability (∆V̇O_2max_) in the HIIT group was significantly associated by the following variables at baseline: V̇O_2max_ (estimate = − 0.53 [− 1.00, − 0.08]), LTP1 (estimate = 0.63 [0.21, 1.03]); and OUES (estimate = 0.45 [0.06, 0.85]). In MICT, albeit not significant, ∆V̇O_2max_ tended to be positively associated with LTP1 (estimate = 0.42 [− 0.14; 0.88], and negatively linked to LTP2 (estimate = − 0.31 [− 0.81; 0.20]).

In the vascular domain, a higher ∆V̇O_2max_ was associated with a lower upper limb StO_2_ slope 2 (estimate = − 0.22 [− 0.38, − 0.04]), and a higher brachial-ankle pulse wave velocity (i.e., higher arterial stiffness) (estimate = 0.32 [0.07, 0.59]) in HIIT; and with a lower limb StO_2_ area under the curve (estimate = − 0.36 [− 0.62, − 0.10]) in MICT.

In the cardiac domain, the HIIT group showed that ∆V̇O_2max_ was significantly positively associated with the left ventricular end-diastolic diameter (LVEDD, estimate = 0.33 [0.00; 0.66]) and negatively associated with left ventricular mass (LV-Mass, estimate = − 0.64 [− 1.17; − 0.09]). In MICT, ∆V̇O_2max_ was associated with right ventricular systolic function (s’ RV, estimate = 0.33 [0.04; 0.66]). In addition, albeit not significant, LV-Mass (estimate = 0.41 [− 0.03, 0.83]) showed a positive association in MICT.

## Discussion

To our knowledge, this was the first study to adequately compare severe- and moderate-intensity exercise adaptations in a group of healthy sedentary individuals. We also comprehensively applied a Bayesian method for classifying responders to exercise training, and we used a Bayesian machine learning method to describe predictors that explain the variability seen in the ∆V̇O_2max_ response to HIIT and MICT. It is important to highlight once again that our exercise training intervention was well-controlled to compare the effects of MICT and HIIT by clearly defining moderate- and severe-intensity exercise regimens, respectively. In addition, we applied a rigorously controlled training stimulus throughout the weeks of training, to ensure that the metabolic stress of the stimulus remained consistent. Our main findings were:

i) HIIT and MICT induced significant increases in V̇O_2max_, with HIIT inducing a significantly greater improvements in V̇O_2max_ compared to MICT. A greater variability in the V̇O_2max_ response classification was observed in the MICT compared to the HIIT group, with approximately 50% of the MICT intervention group classified as “non-responder” and “undecided”;
ii) Our short-term (6 weeks) exercise training intervention could not find any significant morphological changes in the heart as measured by echocardiography. In contrast, small changes in the systolic and diastolic function were found, especially of the right ventricle (i.e., s’ RV);
iii) The variability in the ∆V̇O_2max_ was associated with initial lower cardiorespiratory fitness, higher arterial stiffness, lower left ventricular mass and higher diastolic function (i.e., LVEDD) in HIIT; whereas, lower lower-limb microvascular responsiveness, and higher right ventricular systolic function (i.e., s’ RV) showed a significant association in the MICT group.

### Responders’ classification in the severe- and moderate-intensity domains

Clinical trials are often designed to binarily classify an individual as either responder or non-responder in relation to a given threshold. Such dichotomous approach, however, have its shortcomings. First and foremost, despite the appealing idea to classify the individual trainability to a standard exercise training dose, previous research has not always taken into consideration the usual intraindividual measurement error associated with oxygen uptake measures (Bouchard et al. 2012; Ross et al. 2015; Byrd et al. 2019). In other words, a “responder” could turn into a “non-responder” just because of variability between the measurements. (Mann et al. 2014). Therefore, research rigor requires that such classification takes into account the reproducibility of oxygen uptake measurements (Hecksteden et al. 2015). The ROPE + HDI decision-making method allowed us to run individual statistical tests to then objectively classify each participant based on levels of significance and therefore avoid the dichotomous responder/non-responder classification (Fedorov et al. 2009; Uryniak et al. 2011). Moreover, this full Bayesian framework provided a better error control in comparison with the magnitude-based inference methods (Welsh and Knight 2015; Sainani 2018), which moves the analysis beyond the dichotomization of a continuous measure. It is important to note that, in addition to having a random exercise training allocation, we also performed an ANCOVA, and therefore, excluded the possibility that the regression to the mean phenomenon (i.e., where normal variation in the data can be interpreted as real/meaningful change) was present (Barnett 2004). In this context, the use of Bayesian credible intervals (i.e., HDI) as a decision-making strategy offered a more suitable tool than simply using raw scores, reducing the chance of mathematical coupling (Tu and Gilthorpe 2007; Fountoulakis and Kontis 2012).

Regarding responders to exercise training, a previous study has indicated that individuals that did not respond to moderate-intensity endurance training (i.e., did not significantly increase V̇O_2max_), ended up becoming responders when the exercise dose (i.e., the number of sessions per week) was increased (Montero and Lundby 2017). Specifically, participants who did not respond to the original exercise training intervention were asked to increase the dose of exercise by two extra sessions per week. Interestingly, all participants who had originally been classified as non-responders became responders. However, on Montero & Lundby’s study the exercise intensity was assigned to be 65% of PO_peak_, derived from an incremental test that increased PO by 30 W per minute. As indicated elsewhere (Iannetta et al. 2019, 2020b), it is unlikely that this exercise intensity resulted in a moderate intensity stimulus for the whole sample and, in fact, is most likely to have produced exercise intensities within the heavy or severe domains. Thus, although the authors demonstrated that increasing the number of sessions per week decreased the occurrence of non-responders, the actual metabolic stress from the training intervention is unknown. In relation to this, the present investigation has shown, for the first time, that in the initial phase after starting a structured training program, the actual metabolic stress imposed by the training intervention might be a key modulator in the occurrence of non-responders to exercise. This is reflected by the aforementioned finding that participants who performed six weeks of exercise training within the moderate-intensity domain had a high variability in the V̇O_2max_ response, and half of them were not classified as responders. On the other hand, the majority of the participants of the HIIT group were responders when considering the V̇O_2max_ response to exercise training.

### Cardiovascular markers at PRE associated with ∆V̇O_2max_

Our results showed that participants with increased arterial stiffness and reduced StO_2_ reperfusion rate benefited more from HIIT than MICT, showing a higher ∆V̇O_2max_—see Fig. 5c. Moreover, the training-induced improvement in arterial stiffness (in HIIT) and StO_2_ reperfusion (in MICT) was paralleled by a higher increase of ∆V̇O_2max_. In this regard, the results of the current study strongly agree with previous findings showing that a larger number of healthy sedentary individuals had improvements in arterial stiffness after HIIT than MICT (Ramírez-Vélez et al. 2019). Hemodynamic responses to HIIT may induce greater changes in shear stress, hemodynamic pressure, and circumferential stretch—the main factors contributing to positive structural and functional adaptations of the vasculature—when compared to MICT (Green et al. 2017). However, it is likely that a certain degree of arterial stiffness may be necessary in order be able to detect more significant improvements. Regarding the reperfusion slope findings, even though no previous study has compared the effects of HIIT and MICT on reperfusion slope in healthy individuals, Soares et al. (2019b) showed that 12 weeks of HIIT intervention significantly improved the leg reperfusion slope of coronary heart disease patients. It has been suggested that repetitive transient exposure to some degree of hypoxia within the microvasculature during intense exercise may induce an increase in capillary network. In addition, the expression of genes encoding endothelial proteins associated with increased vascular reactivity to ischemia/reperfusion stimulus (Moreira et al. 2020) may explain the improvements in reperfusion slope. However, similar to the arterial stiffness findings, a certain degree of impairment within the microcirculation may be required to observe significant changes in function. In other words, the participants in the current study were sedentary but clinically healthy. Thus, no large impairments in the microcirculation were found overall—which explains why no significant differences were observed in the StO_2_ reperfusion slopes.

The ∆V̇O_2max_ in the MICT group, on the other hand, showed to be primarily associated with resting right-ventricular systolic function (i.e., s’ RV) at PRE. This finding could have an involvement on the role of the heart performance at a given pre- and after-load, which in turn has a direct association with stroke volume (an increase in contractility will lead to an increase in stroke volume) (Davidson and Giraud 2012). However, we acknowledge that resting cardiac systolic function does not have a direct association with maximal stroke volume. In addition, the fact that participants with a higher right systolic function at rest showed a greater exercise training response in MICT is interesting. This is consistent with previous data indicating the critical role of the right ventricle in the responsiveness to endurance training (Sharma et al. 2015; Bohm et al. 2016; Wasfy and Baggish 2016a, b; Rundqvist et al. 2016; Heiskanen et al. 2016)—the cardiac pumping capacity is considered to be a biological factor influencing cardiorespiratory fitness (Moreira et al. 2020). Left ventricular mass (i.e., LV-Mass) showed opposite associations in HIIT and MICT. While LV-Mass had a negative influence in the ∆V̇O_2max_ (i.e., participants with lower baseline LV-Mass had a greater ∆V̇O_2max_) in HIIT, participants that performed MICT benefited more (i.e., greater ∆V̇O_2max_) if they had a higher baseline LV-Mass (see Fig. 3c for individual changes). Such finding goes in line with the capacity of high-intensity training to induce eccentric hypertrophy—which is directly associated with increases of maximal stroke volume and therefore, V̇O_2max_ (Arbab-Zadeh et al. 2014). However, we must also acknowledge that moderate physical activity has also been shown to induce increases of LV-Mass in a cohort of over a thousand participants (Dawes et al. 2016).

## Limitations

From a statistical perspective, cross-over designs have shown to be a more adequate research design when the main goal is to analyze individual responses to a given intervention (Hecksteden et al. 2015, 2018; Senn 2018). Although the present investigation did not aim to investigate responders rate to exercise training, we would like to stress the fact that we took the following precautions to overcome such limitation: (i) the ROPE + HDI used has an advantage over magnitude-based inference and null-hypothesis testing, such that inferences are made through Bayesian credible intervals (Kruschke 2018; Sainani 2018); (ii) ∆V̇O_2max_ values are subject to random within-subject variation, and for this reason we considered a coefficient of variation of 5.6% around each individual ∆V̇O_2max_, as suggested elsewhere (Hecksteden et al. 2018). Then, we simulated 100 measures deriving a normal distribution for each participant (which the mean of the normal distribution would correspond to the ∆V̇O_2max_, and the standard deviation would correspond to all the possible values around the ∆V̇O_2max_ and the coefficient of variation); and (iii) we considered a conservative value of 20% (the recommended value is 10%) of the pre-training standard deviation as the minimal clinical relevant change in V̇O_2max_ around the null value (i.e., zero) which then the percentage of the Bayesian credible interval within this region was calculated. Finally, the levels of significance, according to the percentage of the credible interval that was within the null region were applied to responders’ classification labels (see Fig. 4).

## Conclusions

In conclusion, our findings highlight the critical influence of exercise-intensity domains on the individual responsiveness to exercise training. Although the variability in the V̇O_2max_ response was similar in both HIIT and MICT (i.e., both groups presented similar ranges in ∆V̇O_2max_), the incidence of responders to exercise in the moderate-intensity group was 52% (11 out of 21 participants); whereas, 95% (20 out of 21 participants) of responders were observed in the group performing exercise training in the severe-intensity domain. In addition, 48% (10 out of 21 participants) were classified as “undecided” or “non-responder” in the moderate-intensity domain training group, whereas 5% (1 out of 21 participants) were classified as “undecided” when training in the severe-intensity domain. The ∆V̇O_2max_ was associated with pre-training measures of arterial stiffness, microvascular responsiveness, left ventricular mass, and right ventricular systolic function. Altogether, these data reinforce the need not only for individualized training prescriptions to avoid the “one size fits all” paradigm in exercise training, but also the need for prescribing exercise intensities based on a model that can somehow account for the actual metabolic stress that is imposed to the system.

## Data Availability

All the data related to the present manuscript are presented in the tables and figures. Additional data can be provided upon reasonable request.

## Abbreviations

[La^−^]: Blood lactate concentration
%PO_peak_: Percentage of peak power output
∆HR/∆V̇O_2_: Heart rate-oxygen uptake relationship
∆V̇O_2max_: Delta of maximal oxygen uptake
ASE: American Society of Echocardiography
BMI: Body mass index
E/A: Ratio between the E-wave (mitral inflow velocity) and the A-wave (atrial inflow velocity)
ECG: Electrocardiogram
EHIS–PAQ: European Health Interview Survey–Physical Activity Questionnaire
ESC: European Society of Cardiology (ESC)
FS: Fractional shortening
HDI: Highest density interval
HR_max_: Maximal heart rate
LTP1: Lactate turning point 1
LTP2: Lactate turning point 2
LV-Mass: Left ventricular mass
LVEDD: Left ventricular end-diastolic diameter
LVMI: Left ventricular mass index
NIRS: Near-infrared spectroscopy
O_2_: Oxygen
OUES: Oxygen uptake efficiency slope
PO_peak_: Peak power output
POST: Post-training
PRE: Pre-training
RER: Respiratory exchange ratio
ROPE: Region of practical equivalence
RVEDD: Right ventricular end-diastolic diameter
StO_2_: Oxygen saturation
TAPSE: Tricuspid annular plane systolic excursion
V̇O_2_: Oxygen uptake
V̇O_2max_: Maximal oxygen uptake
